# Reply: efficacy and safety of CO_2_ laser-assisted sclerectomy surgery for glaucoma: a systematic review and meta-analysis

**DOI:** 10.5935/0004-2749.202100111

**Published:** 2021

**Authors:** Li Dai, Ai-Ling Li, Ling Yu, Jian Ye

**Affiliations:** 1 Department of Ophthalmology, the Affiliated Hospital of Southwest Medical University, Luzhou 646000, Sichuan Province, China; 2 Department of the School of Public Health of Southwest Medical University, Luzhou 646000, Sichuan Province, China; 3 Department of Ophthalmology, Daping Hospital, Army Medical University, Chongqing 400042, China

Dear Editor,

Glaucoma is one of the major causes of blindness worldwide and seriously affects patients’ visual quality and daily life. The most effective measure to prevent glaucomatous damage is to reduce intraocular pressure (IOP) through anti-glaucoma medication, laser treatment, or surgery. CO_2_ laser-assisted sclerectomy surgery (CLASS) is a promising, newly developed surgical treatment applied mainly to the treatment of open-angle glaucoma (OAG). Compared with other filtration surgery, CLASS has unique advantages: requiring only a relatively short learning curve for the surgeon, preventing penetration of the sclera and trabecular meshwork through precise control of laser energy and range, keeping the inner wall of the Schlemm tube intact, and preserving functional trabecular meshwork^(1)^.

As it has been used successfully to treat OAG, related clinical studies on CLASS are increasing year by year. However, most of these are small sample, non-randomized controlled trials (RCT). Further, although most studies argue that CLASS is safe and effective at treating glaucoma, some suggest that CLASS is less potent than trabeculectomy (TRAB) in reducing IOP^(2)^. At present, there is no consensus on whether CLASS is better than TRAB or other filtration surgery.

We evaluated the effectiveness and safety of CLASS for treating glaucoma by systematically reviewing and meta-analyzing related studies as described in table 1. Nine studies were included, three of which compared CLASS with TRAB. Stata15.0 software was used for the statistical analysis.

**Table 1:** Characteristics of the studies included in the meta-analysis

First author, year	Country	Types of Glaucoma	Follow-up eyes	Follow-up period (mo)	Design	Outcome indicators	Jadad scale/ MINORS scores
Ye Z, 2016^(2)^	China	Multiple types	46	3	RCT	①②⑤⑥⑦⑧	5
Liu CH, 2017^(3)^	China	OAG	9	3	non-RCT	⑤⑥⑦⑧	19
Zhang HL, 2018^(4)^	China	Multiple types	76	8-15	non-RCT	①②③④⑤⑥⑦⑧	20
Geffen N, 2016^(5)^	Israel	POAG, PXFG	111	36	non-RCT	⑤⑥⑦⑧	19
Greifner G, 2016^(6)^	Israel	Multiple types	27	24	non-RCT	⑤⑥⑦⑧	20
Jankowska	Poland	POAG, XFG	131	12	non-RCT	①②③④⑤⑥⑦⑧	20
Szmul J, 2018^(7)^							
Sohajda, 2017^(8)^	Hungary	OAG	20	12	non-RCT	④⑤⑥⑦⑧	18
Yick DW, 2016^(9)^	China	Multiple types	23	6	non-RCT	⑤⑥⑦⑧	20
Sohajda Z, 2020^(10)^	Hungary	POAG, PXFG	22	12	non-RCT	④⑤⑥⑦⑧	20

Outcome indicators: ①IOPR% at 1 month postoperative; ②IOPR% at 3 months postoperative; ③IOPR% at 6 months postoperative; ④number of anti-glaucoma medication used 12 months postoperative; ⑤shallow anterior chamber; ⑥choroidal detachment; ⑦hyphema; ⑧iris incarceration.

**Figure 1:**
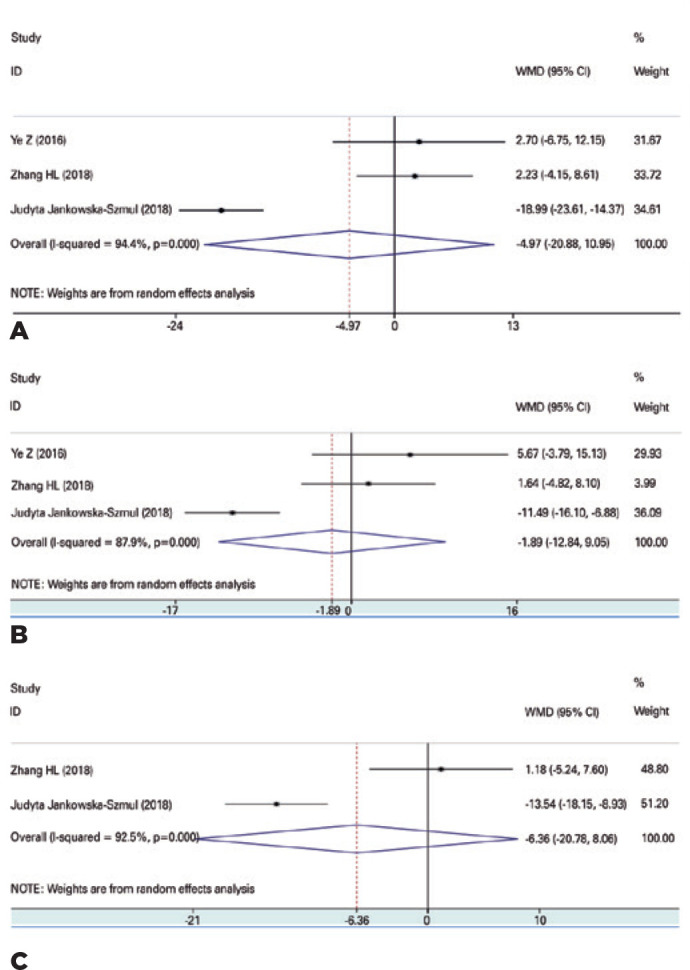
Comparison of IOPR% between CLASS and TRAB at 1, 3, and 6 months after surgery. (A) Comparison of IOPR% between CLASS and TRAB at 1months after surgery. (B) Comparison of IOPR% between CLASS and TRAB at 3 months after surgery. (C) Comparison of IOPR% between CLASS and TRAB at 6 months after surgery.

The meta-analysis showed a statistically significant difference between postoperative and preoperative IOP at 3, 6 and 12 months (MD=-10.95, 95%CI -14.66 ± -7.24, p<0.001, MD=-11.29, 95%CI -16.09 ± -6.50, p<0.001, and MD=-11.14, 95%CI -14.56 ± -7.73, p<0.001, respectively). Three articles compared IOPR% at 1, 3, and 6 months after CLASS and TRAB (Figure 1), and there was no difference at any of these timepoints (MD=-4.97, 95%CI -20.88 ± 10.95, p=0.54, MD= -1.89, 95%CI -12.84 ± 9.05, p=0.74, MD=-6.36, 95%CI -20.78 ± 8.06, p=0.39, respectively). In other words, there was no difference between CLASS and TRAB in the reduction of IOP.

Four studies recorded the average use of anti-glaucoma medication 12 months after CLASS, and the meta-analysis showed that this was reduced compared with preoperative medication use (MD=-1.48, 95%CI -1.95 ± -1.02, p<0.00001). Regarding safety, nine articles reported postoperative complications of CLASS, including shallow anterior chamber, choroidal detachment, hyphema, and iris incarceration. There was a statistically significant difference between CLASS and TRAB in the above postoperative complications, and TRAB group had a higher rate, but no difference in the incidence of iris incarceration (RR=2.40, 95%CI 0.57 ± 10.11, p=0.23). It may be due to the limited data and relatively short follow-up period in this study, or the surgeon’s technique. CLASS was reported to have both high postoperative complete success rate and qualified success rate^(4,7)^. However, as the different definitions in the included studies, we failed to compare it with TRAB.

**Figure 2:**
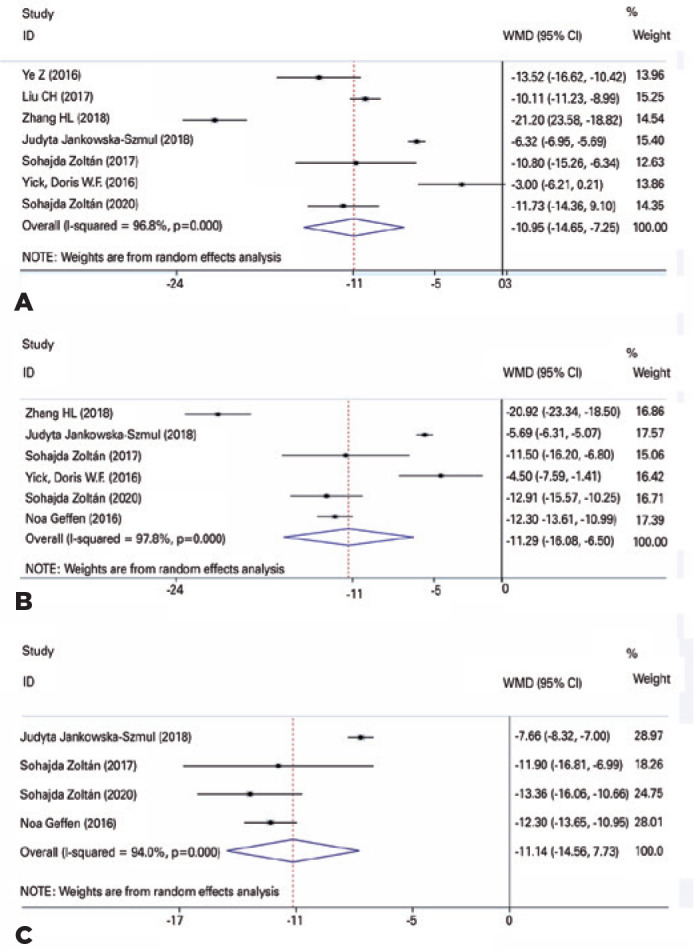
Changes of IOP at 3,6, and 12 months after CLASS. (A) Changes of IOP at 3 months after CLASS. (B) Changes of IOP at 6 months after CLASS. (C) Changes of IOP at 12 months after CLASS

CLASS is increasingly preferred by ophthalmologists for glaucoma treatment. It can effectively reduce the IOP and the use of anti-glaucoma medications and has a good safety profile (Figure 2). However, most of the current studies on CLASS treatment for glaucoma are not RCT. To more accurately assess the effectiveness and safety of CLASS for treating glaucoma, these findings need to be verified by more high-quality RCT with long-term observation and various evaluation indices such as visual acuity and retinal nerve fiber layer thickness.

Since CLASS was introduced into China in 2015, it has been promoted and developed continuously to observe its efficacy combined with other surgical methods in the treatment of glaucoma or glaucoma with cataract. According to Yu et al.^(11)^, CLASS with phacoemulsification may become a safe and effective intervention for patients with primary open-angle glaucoma (POAG) and visually significant cataracts. Zhang et al.^(12)^ also achieved precise treatment for POAG patients in China through the combination of modified CLASS and preventive laser iris management; their findings suggested that this was an effective and safe long-term treatment for POAG. Finally, this meta-analysis showed that CLASS can significantly reduce IOP and has higher safety. We believe that more high-quality RCT studies will confirm the efficacy of CLASS in the future.
